# Gender bias in under-five mortality in low/middle-income countries

**DOI:** 10.1136/bmjgh-2017-000350

**Published:** 2017-07-28

**Authors:** Janaína Calu Costa, Inacio Crochemore Mohnsam da Silva, Cesar Gomes Victora

**Affiliations:** International Center for Equity in Health, Postgraduate Program in Epidemiology, Federal University of Pelotas, Pelotas, RS, Brazil

**Keywords:** Under-five mortality rate, Gender bias, Demographic and Health Surveys

## Abstract

**Introduction:**

Due to biological reasons, boys are more likely to die than girls. The detection of gender bias requires knowing the expected relation between male and female mortality rates at different levels of overall mortality, in the absence of discrimination. Our objective was to compare two approaches aimed at assessing excess female under-five mortality rate (U5MR) in low/middle-income countries.

**Methods:**

We compared the two approaches using data from 60 Demographic and Health Surveys (2005–2014). The prescriptive approach compares observed mortality rates with historical patterns in Western societies where gender discrimination was assumed to be low or absent. The descriptive approach is derived from global estimates of all countries with available data, including those affected by gender bias.

**Results:**

The prescriptive approach showed significant excess female U5MR in 20 countries, compared with only one country according to the descriptive approach. Nevertheless, both models showed similar country rankings. The 13 countries with the highest and the 10 countries with the lowest rankings were the same according to both approaches. Differences in excess female mortality among world regions were significant, but not among country income groups.

**Conclusion:**

Both methods are useful for monitoring time trends, detecting gender-based inequalities and identifying and addressing its causes. The prescriptive approach seems to be more sensitive in the identification of gender bias, but needs to be updated using data from populations with current-day structures of causes of death.

**Key questions**

**What is already known about this topic?**

- Equal mortality rates for girls and boys do not exclude the possibility of gender bias. In the absence of discrimination, higher mortality rates tend to occur among boys due their greater biological frailty.
- The assessment of gender bias in under-5 mortality must consider the fact that boys are more likely to die, and the levels of overall under-5 mortality. Survival advantage of girls is more evident when overall mortality is low.
- Two methods have been used in the literature for this purpose: a prescriptive and a descriptive approach.

**What are the new findings?**

- The two approaches result in similar country rankings according to excess female deaths, but the prescriptive approach identified gender bias in a much larger number of countries than the descriptive approach.
- Both sets of results were very highly correlated but the prescriptive approach tended to show higher excess female mortality than the descriptive one.
- Both approaches suggest that gender bias is more likely in South Asia and Middle East and North Africa.
- There is a weak correlation between excess female under-5 mortality rate (U5MR) and the sex ratio in care-seeking for childhood illness in the same country. Four countries with significant excess female U5MR also had evidence of gender bias in care-seeking.

**Recommendations for policy**

- Both methods are useful to monitor time trends, detect gender-based inequalities and identify and address its causes.
- The prescriptive approach is more sensitive than the descriptive approach, but needs to be updated using present-day structures of causes of death in low/middle-income countries.
- A gender perspective should be an essential aspect of international monitoring health indicators.

## Introduction

Mortality reduction is a key element of the Sustainable Development Goals (SDGs) with a specific goal (SDG 3.2) addressing the mortality of children under 5 years of age.^1^ Disaggregation of child mortality by sex allows the detection of inequalities and, in particular, of systematic gender bias that may result from unfair distribution of resources, discrimination, unequal opportunities or differential treatment for girls and boys.^2–7^

However, assessment of gender bias in child mortality is complex, because equality—that is, equal mortality rates for girls and boys or a sex ratio near to 1—does not imply that there is equity.^7^ Under circumstances where there is no discrimination in healthcare, and where girls and boys have the same access to resources and care, higher mortality rates occur among boys due their greater biological frailty.^5 8^

Such greater frailty of boys has been long recognised. Despite their higher birth weights, newborn boys tend to be less mature than newborn girls and present higher perinatal mortality and more frequent congenital malformations.^9 10^ Poor lung maturation because of male hormones may contribute to the greater vulnerability of male infants to respiratory distress; mortality due to intestinal infections is also usually higher for male children. X-linked immunoregulatory genes contribute to greater resistance to infectious diseases among females.^8 10^ Therefore, equal mortality rates for boys and girls suggest gender bias, as lower mortality rates among girls would be expected.

The sex ratio of child deaths varies according to the overall levels of mortality, since different causes of death may affect girls and boys to different extents. Existing data suggest that the advantage of girls is more evident as overall mortality falls, because perinatal causes and malformations predominate when mortality is low.^4 6 7^ Therefore, adjustment for the overall level of mortality will account for the different structures of causes of death and allow comparison of male and female death rates. A fundamental issue in such analyses is to establish what relation between male and female mortality rates would be the expected in the absence of discrimination, taking into account different levels of mortality rate.^2 7^

Two approaches have been used in the literature to measure the extent of gender inequities in survival, but we were unable to find any comparison of their merits and shortcomings, based on the application of both methods to the same data sets.

The first methodological approach consists in comparing observed sex ratios for mortality with ratios that would be expected in societies where gender discrimination is believed to be low or absent.^7^ This may be referred to as a ‘prescriptive’ approach, such as that used by Hill and Upchurch who relied on records from selected populations of European origin in the 19th and 20th centuries.^7^ Their option to use historical data series is based on the observation that mortality rates in Europe at that time were similar to those currently observed in low/middle-income countries (LMICs).

The second approach is ‘descriptive’ and shows how sex ratios vary in present-day countries with available data, including populations which may be affected by gender bias.^2^ Data sources included vital registration systems, sample registration and surveillance systems, censuses and household surveys. Using a Bayesian model, the descriptive approach estimated the relationship between sex ratios and mortality levels for 195 countries. The model allowed the authors to estimate the expected female mortality rates according to overall levels of under-5 mortality, and to identify countries with outlying sex ratios, when compared with other countries at similar levels of mortality.^2^

In this paper, we compare these two approaches in terms of assessing the presence and magnitude of excess female under-5 mortality rate (U5MR) using representative and standardised data from surveys conducted in LMICs. Based on results from a previous analysis of care-seeking from appropriate healthcare providers in the same countries, we also assess whether countries with excess female mortality present evidence of gender bias in care-seeking.^11^

## Methods

We used data from nationally representative Demographic and Health Surveys (DHS) conducted between 2005 and 2014 in LMICs, which include full birth histories for women of reproductive age. For countries with more than one survey, we selected the most recent one. All surveys with public domain data sets are available on the DHS website (http://dhsprogram.com/).

DHS collect comparable data across countries based on standard model questionnaires.^12^ Women aged 15 to 49 years were asked about their full birth history, including characteristics such as birth dates, sex of the children and survival status. If a child was not alive, the age at death was recorded. From these questions, it is possible to calculate U5MR, or the probability of dying before the fifth anniversary, expressed as the number of deaths per 1000 live births.

A synthetic cohort life table approach was used, in which the probabilities of death from the beginning to the end of narrow age segments (0–30 days and 1–2, 3–5, 6–11, 12–23, 24–35, 36–47, 48–59 complete months) are calculated based on the actual cohort mortality experience; these are later combined to estimate the U5MR for the whole national samples and separately for boys and girls.^12 13^

We employed a jackknife method to calculate the variance of the estimates from the DHS data sets. In this procedure, estimates are calculated from repeated subsamples formed by deleting one sampling unit from the original sample at a time.^13^ The distribution of the estimates made from these multiple subsamples is used to characterise the sampling variability. This procedure allowed us to calculate standard errors and 95% CI.

DHS also included questions for mothers or caretakers about care-seeking for children under 5 years of age who experienced diarrhoea, fever or suspected pneumonia on the 2 weeks prior to the interview. We used the same data sets to calculate a composite care-seeking indicator which represents the proportion of children with any of these conditions who were taken to an appropriate provider.^11^ This was estimated separately for girls and boys in each survey, and the sex ratio was calculated dividing the proportion of girls by the proportion of boys taken to an appropriate healthcare provider.

To identify countries with excess female mortality, the observed estimates (calculated from DHS) were compared with the expected female U5MR resulting from two different statistical models.

The first approach proposed by Hill and Upchurch,^7^ and here defined as ‘prescriptive’, was developed from life tables covering the period between 1820 and 1964, which are believed to have high quality, provide long time series and cover a wide range of overall mortality levels.^7^ The data are from Northwestern European countries or populations (England and Wales, France, the Netherlands, New Zealand and Sweden) where it is assumed that gender bias against girls have been relatively modest or absent, and stable over time.^7^ With these data, an empirical standard of female advantage was obtained by fitting a smooth curve, using a locally weighted least squares procedure. This curve was used to predict the expected sex ratio, given a level of male U5MR. Expected female U5MR was calculated by multiplying the female/male sex ratio by the value of male U5MR.

The ‘descriptive’ approach, developed by Alkema *et al*,^2^ used data available from multiple sources in 195 countries since 1950.^2^Penalised B-splines regression (flexible regression model) was applied to determine the so-called ‘global relation’ between the sex ratio and the total mortality rate separately for infant (<1 year) and child (1–4 years) mortality rates.^2^ These rates were later combined as the U5MR, and excess female mortality rates were obtained. The authors modelled country-specific sex ratios using the product of the expected sex ratio (based on regression model) and a country-specific multiplier, which represents the relative advantage or disadvantage of girls to boys compared with other countries at similar total mortality rates.^2^ Country-specific average levels were determined using a Bayesian hierarchical model, allowing for outlying countries where greater male or female advantages might be seen.^2^ The global relation between expected sex ratios and total mortality rates for age groups implies that for each value of male mortality, there exists an associated value of expected female mortality, such that the ratio of male mortality over expected female mortality is equal to the expected ratio at the implied level of total mortality.^2^ Unpublished tabulations of the expected female U5MR according to the overall U5MR were kindly provided by the authors (Alkema L, personal communication). In table 1, we compare the two approaches according to its methodologies.

**Table 1:** Comparison between the two approaches used to estimate excess female under-5 mortality

	Hill and Upchurch^7^	Alkema *et al*^2^
Type	Prescriptive	Descriptive
Data	Historical series from four Northwestern European countries (England and Wales, France, the Netherlands and Sweden) and New Zealand, between 1820 and 1964	All available data from vital registration systems, sample registration and surveillance systems, surveys and censuses from 195 countries since 1950.
Age ranges	Infant (<1 year), child (1–4 years) and under 5 (<5 years)	
	Intended to reflect sex differentials in childhood mortality in the absence of substantial discrimination, but at relatively high levels of child mortality. Described by the authors as a mortality ‘standard’.	Intended to reflect actual sex ratios in mortality, regardless of the presence of gender bias. Country-specific sex ratios are presented; these results are based on the product of the expected sex ratio and a country-specific multiplier, which represents the relative advantage or disadvantage of girls to boys compared with other countries with similar total mortality rates.
Original estimates		
Assessment of gender bias	Female advantage index (difference between the observed and expected female/male mortality ratios) for any given level of male mortality	Countries with outlying sex ratios. Excess female mortality expressed as the difference between the expected and estimated female mortality rates

Estimation of excess female mortality rate by country was obtained from the comparison between the observed female U5MR and the expected values resulting from both approaches, by the following formula:

$$\left[\frac{observed\;female\;U5MR}{expected\;female\;U5MR}-1\right]\times 100$$

Excess female U5MR was chosen to express the presence and magnitude of gender bias because it is easier to interpret than sex ratio (which can also be calculated from the proposed approaches). The excess is presented for each country as percentage; positive values suggest excess female mortality, indicating gender bias against girls. We assessed statistical significance by checking whether the expected values were included in the 95% CI of observed female U5MR.

Descriptive statistics for the excess female U5MR resulting from the comparison to both models were calculated. Correlation between the results from the two approaches were analysed by Pearson's coefficient as well as by linear regression. Countries were ranked by values of excess female U5MR obtained from each model to compare them.

We used analysis of variance (ANOVA) to compare the two estimates of excess female U5MR according to world regions (based on Unicef classification) and country income groups (low-income, lower middle-income and upper middle-income, based on 2012 World Bank classification).^14 15^

Finally, in order to assess whether care-seeking practices are associated with gender bias in U5MR, values of excess female mortality were correlated with the sex ratio in care-seeking using Pearson's coefficient of correlation. Countries with significant gender bias in care-seeking were compared with those with significant excess female U5MR.

Analyses were conducted using Stata V.13.1 (StataCorp) and accounted for the sampling design of each survey. The study was based on publicly available data and ethical clearance was the responsibility of the institutions that administered the surveys.

## Results

Data were available for 60 countries where the most recent DHS took place since 2005 (median 2012). The overall U5MR ranged from 18.7 to 174.7 deaths per 1000 live births; male U5MR from 21.3 to 185.4, and female U5MR from 13.4 to 163.7.

Table 2 presents the observed female U5MR with 95% CI by country, and includes expected values and the excess female U5MR resulting from both prescriptive and descriptive approaches.

**Table 2:** Observed values from DHS and expected and excess female mortality rates according to prescriptive (Hill and Upchurch) and descriptive (Alkema *et al*) approaches

		Observed (DHS)			Prescriptive approach		Descriptive approach	
Country	Year	Total U5MR	Male U5MR	Female U5MR (95% CI)	Expected female U5MR	Excess female U5MR	Expected female U5MR	Excess female U5MR
Albania	2008	21.9	27.3	16.1 (9.7 to 22.4)	21.3	−24.6%	19.6	−18.0%
Armenia	2010	21.5	21.3	21.7 (11.7 to 31.8)	16.5	31.9%	19.6	11.0%
Azerbaijan	2006	57.3	64.3	49.0 (36.8 to 61.2)	51.6	−5.0%	52.1	−6.0%
Bangladesh	2014	53.7	51.8	55.7 (48.2 to 63.3)*	41.1	35.6%	49.2	13.2%
Benin	2011	75.0	78.8	70.8 (64.8 to 76.8)*	63.8	11.0%	69.5	2.0%
Bolivia	2008	75.2	79.1	71.1 (63.5 to 78.7)	64.1	11.0%	69.5	2.4%
Burkina Faso	2010	146.9	152.6	141.0 (132.5 to 149.5)*	129.5	8.9%	139.3	1.2%
Burundi	2010	125.1	134.1	116.0 (105.5 to 126.4)	112.6	3.0%	117.9	−1.7%
Cambodia	2014	47.4	53.3	41.2 (34.3 to 48.0)	42.4	−2.9%	42.6	−3.5%
Cameroon	2011	128.1	134.8	121.3 (111.6 to 131)	113.3	7.1%	120.8	0.3%
Chad	2014	147.2	154.7	139.3 (128.3 to 150.4)	131.4	6.0%	139.3	0.0%
Colombia	2010	21.6	23.8	19.3 (16.7 to 21.8)	18.5	4.2%	19.6	−1.5%
Comoros	2012	49.7	47.8	51.8 (40.7 to 62.9)*	37.8	36.8%	45.5	13.9%
Congo Brazzaville	2011	80.7	84.4	76.9 (67.3 to 86.4)	68.6	12.0%	75.3	2.1%
Congo Democratic Republic	2013	111.0	113.9	108.1 (100.4 to 115.9)*	94.5	14.4%	104.4	3.6%
Cote d'Ivoire	2011	115.5	132.8	97.4 (87.2 to 107.7)*†	111.4	−12.6%	109.2	−10.8%
Dominican Republic	2013	33.8	34.2	33.3 (25.0 to 41.7)	26.8	24.6%	30.4	9.5%
Egypt	2014	30.3	30.4	30.1 (26.3 to 34.0)*	23.7	27.0%	26.8	12.5%
Ethiopia	2011	109.5	120.8	97.4 (87.4 to 107.5)	100.7	−3.2%	102.4	−4.9%
Gabon	2012	63.2	70.9	55.9 (45.9 to 65.9)	57.1	−2.0%	57.8	−3.3%
Gambia	2013	60.8	63.3	58.2 (49.7 to 66.8)	50.7	14.8%	55.9	4.2%
Ghana	2014	69.9	77.4	62.1 (54.8 to 69.4)	62.6	−0.8%	64.6	−3.8%
Guinea	2012	132.3	141.4	122.9 (109 to 136.8)	119.2	3.1%	124.8	−1.5%
Guyana	2009	39.8	40.6	39.0 (28.9 to 49.0)	32.0	21.8%	36.0	8.2%
Haiti	2012	92.4	104.0	80.3 (72.1 to 88.5)	85.7	−6.3%	85.9	−6.5%
Honduras	2011	29.4	29.3	29.4 (25.5 to 33.4)*	22.9	28.8%	25.9	13.7%
India	2005	84.9	81.8	88.2 (84.2 to 92.1)*†	66.4	32.7%	79.1	11.4%
Indonesia	2012	42.4	47.9	36.7 (32.9 to 40.6)	37.9	−3.1%	37.9	−3.1%
Jordan	2012	20.2	21.4	19.0 (13.8 to 24.1)	16.6	14.2%	17.8	6.6%
Kenya	2014	55.5	58.7	52.2 (47.5 to 56.9)*	46.8	11.5%	50.2	4.0%
Kyrgyzstan	2012	32.5	31.5	33.5 (25.9 to 41.0)*	24.6	36.2%	28.6	17.0%
Lesotho	2014	91.7	101.5	81.9 (69.1 to 94.7)	83.5	−2.0%	85.9	−4.7%
Liberia	2013	111.2	112.3	110.0 (96.7 to 123.4)*	93.1	18.2%	104.4	5.4%
Madagascar	2008	81.5	84.8	78.0 (70.5 to 85.5)*	69.0	13.0%	75.3	3.6%
Malawi	2010	126.1	136.5	115.8 (109.4 to 122.2)	114.7	0.9%	118.9	−2.6%
Maldives	2009	26.9	28.7	25.0 (16.9 to 33.1)	22.3	11.9%	24.1	3.8%
Mali	2012	104.0	116.5	90.7 (81.9 to 99.5)	96.8	−6.3%	97.5	−7.0%
Moldova	2005	26.3	32.5	19.7 (12.2 to 27.2)	25.4	−22.6%	23.2	−15.1%
Mozambique	2011	108.0	113.2	102.8 (94.9 to 110.8)*	93.9	9.5%	101.4	1.4%
Namibia	2013	58.9	63.6	54.4 (47.1 to 61.7)	50.9	6.9%	54.0	0.8%
Nepal	2011	62.2	62.2	62.2 (53.9 to 70.6)*	49.7	25.1%	56.9	9.4%
Niger	2012	151.7	158.7	144.6 (135 to 154.1)	135.1	7.0%	144.2	0.3%
Nigeria	2013	142.9	149.6	136.0 (127.6 to 144.3)*	126.8	7.3%	135.5	0.4%
Pakistan	2012	96.4	96.9	95.9 (86.9 to 104.8)*	79.5	20.6%	89.8	6.8%
Peru	2012	25.1	26.9	23.3 (19.4 to 27.1)	20.9	11.4%	22.3	4.6%
Philippines	2013	32.1	33.7	30.4 (25.5 to 35.3)	26.3	15.4%	28.6	6.3%
Rwanda	2014	65.3	68.0	62.5 (56.1 to 69)*	54.6	14.4%	59.8	4.6%
Sao Tome and Principe	2008	70.5	85.8	54.5 (40.7 to 68.4)*	69.9	−21.9%	65.6	−16.8%
Senegal	2014	61.9	62.6	61.3 (49.4 to 73.1)	50.1	22.3%	56.9	7.8%
Sierra Leone	2013	174.7	185.4	163.7 (153.7 to 173.7)	159.9	2.4%	166.6	−1.7%
Swaziland	2006	106.4	108.7	103.9 (91.0 to 116.7)*	89.9	15.6%	99.5	4.4%
Tajikistan	2012	47.9	50.6	45.1 (37.2 to 53.0)	40.1	12.5%	43.6	3.6%
Tanzania	2010	92.1	96.5	87.6 (78.5 to 96.7)	79.2	10.6%	85.9	2.0%
Timor-Leste	2009	79.7	83.4	75.8 (69.2 to 82.5)*	67.8	11.9%	74.3	2.1%
Togo	2013	93.2	99.1	87.1 (78.5 to 95.7)	81.4	6.9%	86.9	0.2%
Uganda	2011	105.5	113.5	97.4 (87.5 to 107.2)	94.1	3.4%	98.5	−1.2%
Ukraine	2007	18.7	23.4	13.4 (6.8 to 20.0)	18.2	−26.2%	16.9	−20.6%
Yemen	2013	57.1	58.5	55.7 (50.4 to 60.9)*	46.7	19.2%	52.1	6.8%
Zambia	2013	79.8	86.5	72.9 (66.8 to 79.1)	70.5	3.5%	74.3	−1.8%
Zimbabwe	2010	78.0	87.6	68.4 (60.1 to 76.7)	71.4	−4.2%	72.3	−5.5%

*CI does not include the expected value according to prescriptive approach. Excess female U5MR=[(observed/expected)−1]×100.

†CI does not include the expected value according to descriptive approach

DHS, Demographic and Health Surveys; U5MR, under-5 mortality rate.

Excess female mortality was markedly higher for the prescriptive (mean=8.7%; SD=14.3; range from −26.2% in Ukraine to 36.8% in Comoros) than for the descriptive approach (mean=1.2%; SD=7.7; range from −20.6% in Ukraine to 17.0% in Kyrgyzstan).

Comparison of the observed and expected values according to the prescriptive approach shows that in 20 countries, the 95% CI of the observed female U5MR did not include the expected values: Bangladesh, Benin, Burkina Faso, Comoros, Congo Democratic Republic, Egypt, Honduras, India, Kenya, Kyrgyzstan, Liberia, Madagascar, Mozambique, Nepal, Nigeria, Pakistan, Rwanda, Swaziland, Timor-Leste and Yemen. In two countries, the observed value was significantly lower than the expected female mortality rate: Côte d’Ivoire and São Tomé and Príncipe.

Comparisons to the descriptive approach showed only two significant differences: in India, the observed female mortality rate was significantly higher than expected, and in Côte d’Ivoire it was significantly lower.

**Figure 1:**
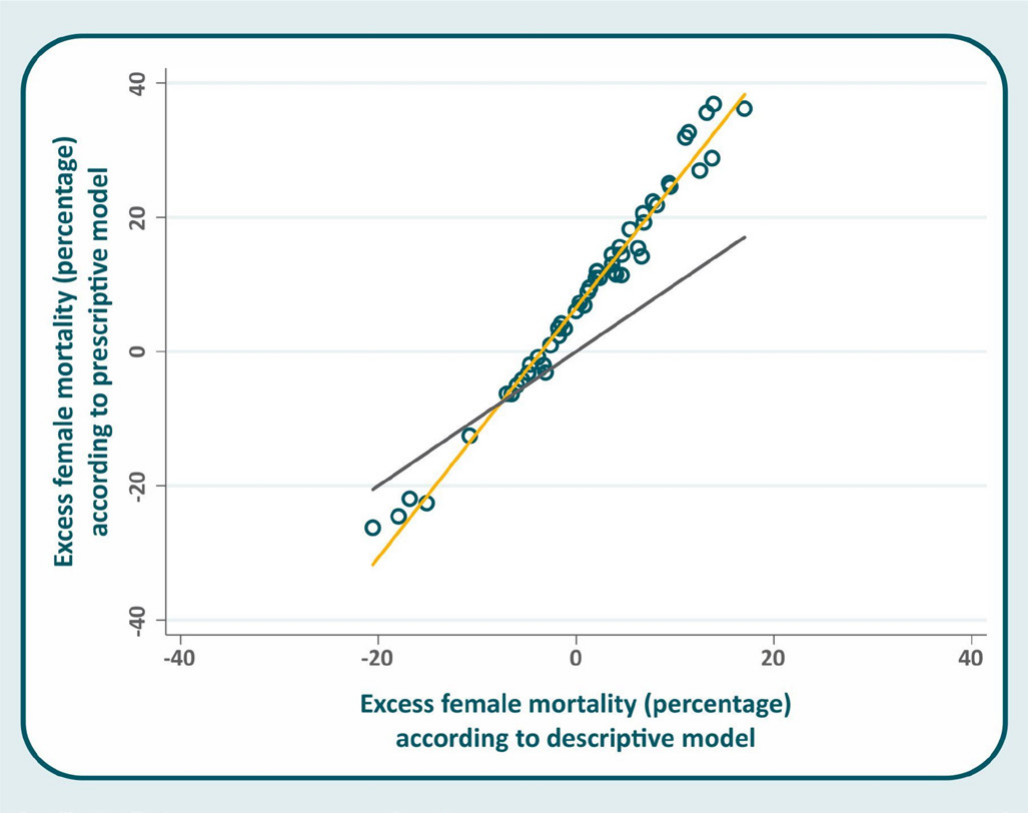
Scatter diagram of excess female mortality according to the prescriptive and descriptive approaches (the black line represents perfect correlation; the yellow line shows fitted values).

Although excess female mortality varied substantially from one approach to the other, both sets of results were very highly correlated (figure 1; Pearson's r=0.989; p<0.001). However, the slope of the regression line was markedly different from one (β=1.84; 95% CI 1.77 to 1.91)—which would indicate perfect equality—confirming that the prescriptive approach tended to show higher excess female mortality than the descriptive one.

When ranking countries by the values of excess female U5MR, we found that the 13 countries with the highest and the 10 countries with the lowest rankings were the same according to both approaches (table 3). It should be noted that the rankings are based on the point estimates for excess female mortality, and for some of the high/low-ranked countries, the CI for the observed female U5MR included the expected value. These results were based on the observed/expected ratios. When countries were ranked according to the absolute difference (in deaths per 1000) between the observed and expected female U5MR, again both models showed agreement regarding nine of the 10 countries with the highest excess of female deaths (data not shown).

**Table 3:** Countries with the highest and lowest rankings of excess female mortality according to the two approaches (countries were ordered according to the ranking position of the prescriptive approach)

Country	Prescriptive approach		Descriptive approach	
	Rank	Excess female U5MR*	Rank	Excess female U5MR*
Thirteen countries with highest rankings				
Comoros	1	36.8%†	2	13.9%
Kyrgyzstan	2	36.2%†	1	17.0%
Bangladesh	3	35.6%†	4	13.21%
India	4	32.7%†	6	11.4%‡
Armenia	5	31.9%	7	11.0%
Honduras	6	28.8%†	3	13.7%
Egypt	7	27.0%†	5	12.5%
Nepal	8	25.1%†	9	9.4%
Dominican Republic	9	24.6%	8	9.5%
Senegal	10	22.3%	11	7.8%
Guyana	11	21.8%	10	8.2%
Pakistan	12	20.6%†	13	6.8%
Yemen	13	19.2%†	12	6.8%
Ten countries with lowest rankings				
Ethiopia	51	−3.2%	51	−4.8%
Zimbabwe	52	−4.2%	52	−5.5%
Azerbaijan	53	−5.0%	53	−6.0%
Mali	54	−6.3%	55	−7.0%
Haiti	55	−6.3%	54	−6.5%
Cote d'Ivoire	56	−12.6%†	56	−10.8%‡
São Tome and Principe	57	−21.9%†	58	−16.8%
Moldova	58	−22.6%	57	−15.1%
Albania	59	−24.6%	59	−18.0%
Ukraine	60	−26.2%	60	−20.6%

*Excess female U5MR=[(observed/expected)−1]x100.

†Confidence interval does not include the expected value according to the prescriptive approach.

‡Confidence interval does not include the expected value according to the descriptive approach.

U5MR, under-5 mortality rate.

Table 4 shows excess female U5MR by world regions and income groups. In South Asia and in the Middle East and North Africa regions, all countries presented positive values, both for the prescriptive and descriptive approaches. The tests for heterogeneity among regions were statistically significant (ANOVA, p values 0.032 and 0.025, respectively for the prescriptive and descriptive results). Differences among country income groups were not significant for either model (p=0.61 and 0.72). A test for linear trend based on the three income groups was also non-significant (p=0.35 and 0.42).

**Table 4:** Mean, maximum and minimum values of excess female U5MR* according to prescriptive and descriptive approaches by world region and country income groups

	Countries (n)	Excess female U5MR (%)					
		Prescriptive approach			Descriptive approach		
		Mean	Minimum	Maximum	Mean	Minimum	Maximum
World region							
CEE and CIS**	7	0.3	−26.2	36.2	−4.0	−20.6	17.0
East Asia and Pacific	4	5.3	−3.1	15.4	0.5	−3.5	6.3
Eastern and Southern Africa	15	8.0	−4.2	36.8	0.8	−5.5	13.9
Latin America and Caribbean	7	13.6	−6.3	28.8	4.3	−6.5	13.7
Middle East and North Africa	3	20.1	14.2	27.0	8.7	6.6	12.5
South Asia	5	25.2	11.9	35.6	8.9	3.8	13.2
West Central Central Africa	19	5.1	−21.9	22.3	−0.9	−16.8	7.8
Income group							
Low-income	28	10.1	−6.3	36.8	1.9	−7.0	17.0
Lower-middle income	23	8.6	−26.2	32.7	0.85	−20.6	13.7
Upper-middle income	9	4.6	−24.6	24.6	−0.4	−18	9.5

*U5MR, under-5 mortality rate; **CEE and CIS, Central and Eastern Europe and the Commonwealth of Independent States.

**Figure 2:**
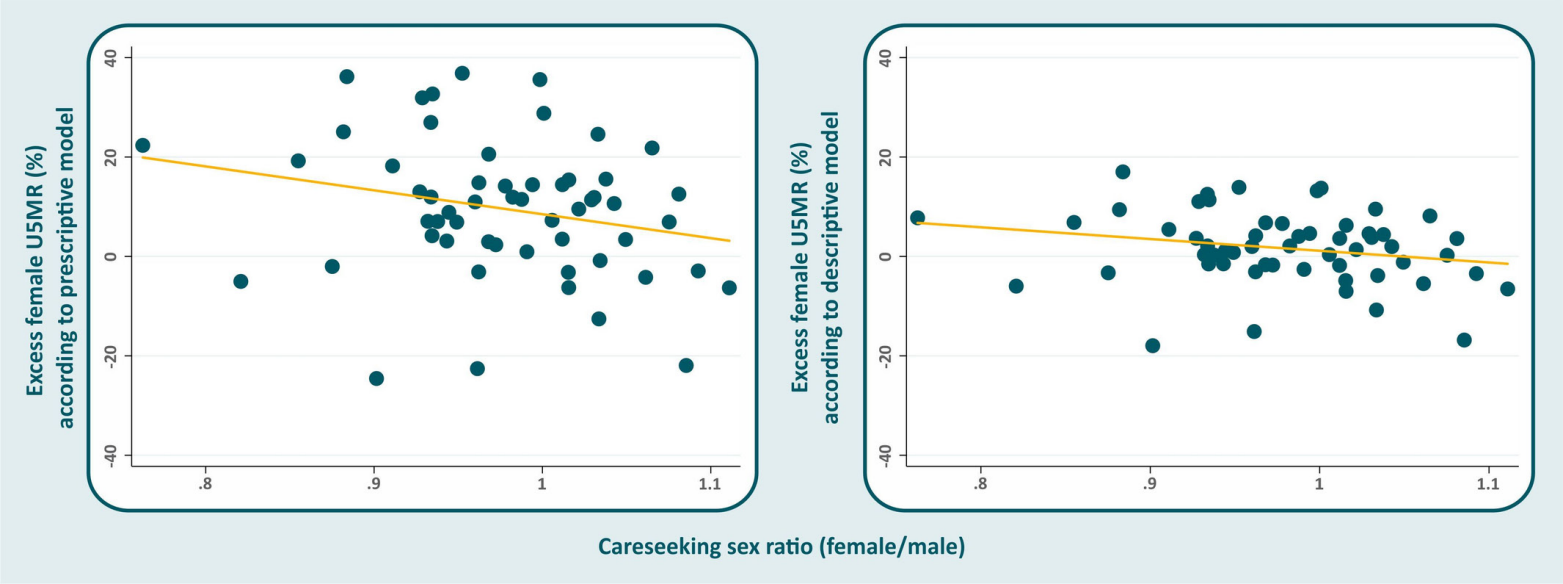
Scatter diagram and line of fitted values of excess female under-5 mortality rate (U5MR) in the prescriptive (left) and descriptive (right) approaches, according to the female/male care-seeking ratio for common childhood conditions.

Data on care-seeking were available for 57 of the 60 countries. figure 2 shows weak inverse correlations, as expected, between care-seeking sex ratios and excess female mortality according to the prescriptive (Pearson's r=−0.237; p=0.078) and descriptive (r=−0.221; p=0.101) approaches, but the associations were not significant. We identified four countries with significant excess female U5MR and in which girls are less likely than boys to be taken to an appropriate provider: India, Egypt, Liberia and Yemen.

## Discussion

The assessment of gender bias in U5MR should not be solely based on the observed difference between estimates for boys and girls. One needs to take into account the higher biological risk of boys, compared with girls.

Our results suggest that the two approaches proposed for assessing excess female mortality present similarities in ranking countries according to the degree of inequality, but differ on assessing its magnitude. The higher magnitude of inequalities obtained with the model proposed by Hill and Upchurch is related to the prescriptive nature of their approach, in which mortality ratios are compared with populations where gender bias is assumed to be low or absent. In contrast, the model proposed by Alkema *et al* adopts a descriptive approach, as the authors avoid a judgement on discrimination and compare countries to a global pattern, that includes countries where gender discrimination may exist.^2 7^

In their original analyses, Alkema *et al* applied a Bayesian model to estimate the global relation between total under-5 mortality level and sex ratios. They identified 10 countries with excess female mortality in 2012: Afghanistan, Bahrain, Bangladesh, China, Egypt, India, Iran, Jordan, Nepal and Pakistan.^2^ Six of these 10 countries had DHS data sets available in the period assessed in the present study. We found significant excess female U5MR in five of them according to the prescriptive model (Bangladesh, Egypt, India, Nepal and Pakistan), but the descriptive model resulted in a significant excess only in India, which was therefore, the only country where excess female mortality was identified by both models. In India, gender-based discrimination has deep social and cultural roots which have been the subject of several studies.^16–20^

Alkema *et al* attribute part of the differences between the two approaches to different sources of data: they use recent data from 195 countries, while Hill and Upchurch analysed only five purposefully selected countries.^2^ The authors also argue that immunisation can result in a decrease in sex ratios of mortality in countries with high death rates, leading to lower sex ratios at present than were the case in the older data sets used by Hill, for the same overall mortality levels.^2^ The authors cite the work of Aaby *et al* in West Africa, suggesting that DTP—diphtheria, tetanus, and pertussis—vaccination can increase mortality rates for girls, whereas measles vaccination reduces female mortality.^21^

Differences among world regions were significant, and our results suggest that gender bias is more likely in South Asia and Middle East and North Africa. These results should be interpreted with caution because not every country in any region has recent DHS surveys. Also, we opted not to weigh the data by population because data were not available for some countries in every region.

Both models indicate that female advantage increases as total mortality decreases.^2 7^ Low mortality rates include a high proportion of early neonatal deaths due to perinatal conditions and malformations, which tend to be more frequent among boys and are also more difficult to prevent.^5 6 8 10^

The Alkema *et al* approach predicts that female advantage will start to decline when U5MR is below 20 per thousand, an observation that had been made earlier for high-income countries.^6^ Only one country included in our analyses presented such low levels (Ukraine, with 18.7/1000 live births).

Excess female mortality can be due to discrimination by gender, especially in nutrition and healthcare. In an earlier set of analyses, we estimated gender bias in care-seeking for common childhood illnesses or symptoms, based on the same DHS data sets used in the present analyses.^11^ Significant differences in care-seeking for sick boys and girls were not observed in most countries. However, we found evidence of gender bias in four countries that were also identified in the present set of analyses with significant excess female U5MR: India, Egypt, Liberia and Yemen. A recent Unicef study investigated sex differences in care-seeking by type of provider in children aged less than 5 years.^22^ Based on 67 DHS, the overall result was consistent with our analyses: there was no evidence of gender bias in most countries, but there were specific locations where bias was likely.^22^

The limitations of our analyses include the use of retrospective data collected from national surveys. Estimates of mortality rates from self-reported reproductive histories may underestimate actual rates. Misreporting of child birth dates, if systematic across sampled birth stories, can lead to bias in estimates of U5MR.^13^ In their original article, Hill and Upchurch raise the possibility that omission of deaths children may vary according to the child's sex and thus bias sex ratios, but found no evidence of such bias in their data sets.^7^ In addition, small sample sizes in some surveys—particularly for a relatively rare outcome such as mortality—may decrease the statistical power of the comparisons and lead to non-significant results. For example, a relatively small excess death ratio may be detected as statistically significant in a country with a large survey such as India, whereas the same excess would not be significant in other countries with smaller surveys.

Our findings suggest that a descriptive approach may fail to identify countries where there is gender bias. On the other hand, the prescriptive approach has limitations, particularly due to its reliance on data from a period in the past when causes of death were not the same as those in present-day LMICs. Further methodological work on the assessment of gender bias is urgently needed.

The recently proposed SDGs place gender issues at the centre of national development. Specifically, goal 17.18 requires disaggregation of national statistics by sex.^1^ Detecting and quantifying the excess deaths of girls still is a methodological challenge. Applying the proposed approaches to existing and easily accessible data, such as DHS, led to similar conclusions regarding the ranking of countries in terms of excess female deaths, but to different estimates of the magnitude of gender bias.

For the time being, we recommend that both methods should be used to monitor time trends, to detect gender-based inequalities and to identify and address its causes. The prescriptive approach appears to be more sensitive than the descriptive approach in identifying countries with excess female mortality, but work is needed to update the prescriptive model to reflect present-day causes of deaths in LMICs.
